# Chromosome-Level Genome Assembly and Annotation of the *Cronartium ribicola* Strain LQ, an Important Fungal Forest Pathogen from China

**DOI:** 10.3390/jof12070471

**Published:** 2026-06-26

**Authors:** Hairui Wang, Qingyi Zhao, Xiong Xiong, Quan Lu, Zhongdong Yu, Danlei Li, Zixuan Kong, Jingwen Sun, Jiawei Liu, Yehan Tian, Huixiang Liu

**Affiliations:** 1 College of Plant Protection, Shandong Agricultural University, Tai’an 271018, China; 2 Ecology and Nature Conservation Institute, Chinese Academy of Forestry, Beijing 100091, China; 3 College of Forestry, Northwest A&F University, Yangling 712100, China; 4 College of Forestry, Northeast Forestry University, Harbin 150006, China

**Keywords:** *Cronartium ribicola*, whole genome, CAZymes, secondary metabolites, effector proteins

## Abstract

*Cronartium ribicola* is a globally distributed fungal pathogen that infects *Pinus armandii* Franch., causing widespread tree mortality. In this study, we report a chromosomal-level genome assembly of *C. ribicola* LQ (262.51 Mb, N50 = 15.4 Mb, GC content = 38.4%) using integrated Illumina short-read, PacBio long-read, and Hi-C sequencing technologies. Ultimately, a total of 94.83% of the assembled sequences were anchored onto 17 pseudo-chromosomes. Genome annotation predicted 10,222 protein-coding genes, and repetitive sequences accounted for 91.67% of the genome. Benchmarking Universal Single-Copy Ortholog (BUSCO) analysis demonstrated 94.03% genome completeness, with functional annotations covering 88.32% of the genes. A total of 352 carbohydrate-active enzyme (CAZyme) genes, predominantly glycoside hydrolases (45.17%), and 8 secondary metabolite biosynthetic gene clusters were identified, indicating strong host tissue degradation capability and diverse virulence factors. Overall, this study provides valuable genomic resources for dissecting the pathogenicity, host interaction mechanisms, and resistance gene discovery of *C. ribicola* and establishes a foundation for developing virulence-targeted disease control strategies.

## 1. Introduction

*Cronartium ribicola* is the pathogenic fungus that causes pine blister rust, posing a serious threat to global forest ecosystems and the forestry industry. It can infect important forest tree species such as *Pinus strobus* Linn., *P. lambertiana* Douglas, *P. armandii* Franch., and *P. koraiensis* Siebold & Zucc., leading to canker formation on branches and trunks, growth inhibition, and widespread death of pine trees [1,2,3]. In recent years, *Pinus armandii* blister rust caused by *C. ribicola* has shown a trend of increasing severity in China, becoming a major biological disaster threatening the sustainable and healthy development and ecological security of Armandii pine forests. Therefore, *C. ribicola* is an important target for forest disease management.

Although fungicides such as triazolone and chlorothalonil have been widely applied to manage *C. ribicola*, their long-term and extensive use has led to increasing concerns regarding environmental contamination and the emergence of fungicide-resistant strains [4]. Biological control approaches, particularly those employing antagonistic microorganisms such as *Trichoderma* spp., have demonstrated promising potential in suppressing disease development. However, their effectiveness under field conditions is often variable and may not be universally reliable [5]. In addition, the wide alternate host spectrum of the pathogen, together with its high ecological adaptability, poses significant challenges to the development of reliable and sustainable disease management strategies.

In recent years, advances in high-throughput sequencing technologies have greatly accelerated research on fungal pathogens by enabling comprehensive genomic investigations. Genome-wide analyses have identified numerous virulence-associated gene families in plant-pathogenic fungi, such as glycoside hydrolases, peptidoglycan-degrading enzymes, and secondary metabolite biosynthetic clusters, which play key roles in host infection and tissue colonization [6,7]. The increasing availability of genomic resources has also supported the identification of potential molecular targets for disease control, particularly genes indispensable for fungal growth, virulence, and persistence [8]. Although studies have reported the genome of *C. ribicola* 11-2 and Cypress_4 v1.0, the pathogens that cause white pine blister rust [9,10], severe sequence fragmentation and genome incompleteness (genome sizes of 94.3 Mb and 211.63 Mb, respectively) have hindered in-depth research into the pathogen. To date, no high-quality genome sequences of *C. ribicola* from China has been reported, representing a significant gap in both fundamental and applied research.

Here, we assembled a Chromosomal-level genome of *C. ribicola* LQ using an integrated approach that combined PacBio long-read sequencing, Illumina short-read sequencing, and Hi-C sequencing technologies. The quality of genome assembly and annotation was assessed by examining assembly continuity using the N50 metric as well as the completeness of conserved protein-coding genes. Functional characterization of predicted proteins was subsequently conducted through sequence comparisons against several public databases, including Nr, SwissProt, KEGG, and KOG. Overall, this study offers a valuable genomic resource that supports further investigations into the pathogenic mechanisms, genetic variation, and evolutionary dynamics of *C. ribicola*.

## 2. Materials and Methods

### 2.1. Fungal Strain

**Figure 1:**
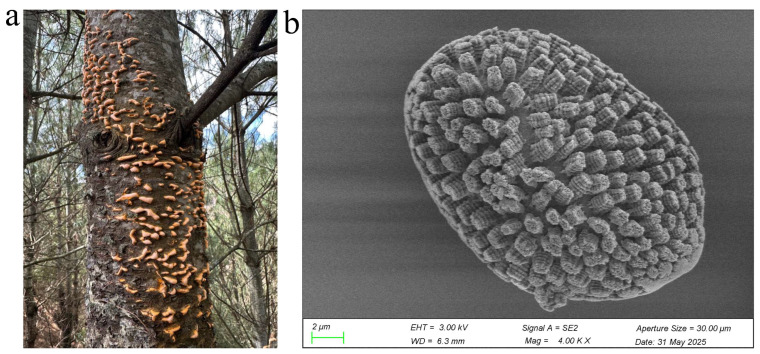
Disease symptoms caused by *Cronartium ribicola* strain LQ in the field. (**a**): *Pinus armandii* infected with *C. ribicola* LQ; (**b**): Morphology of *C. ribicola* LQ under scanning electron microscopy. The aeciospores are ovoid, with a verrucose surface ornamentation.

The *Cronartium ribicola* strain LQ (26.15° N, 102.53° E) (Figure 1a,b), isolated from Luquan County, Yunnan Province, China, is preserved in the Key Laboratory of Agricultural Microbiology, Shandong Agricultural University, China. To minimize the risk of airborne contamination and cross-contamination among aecia, canker samples were collected before the aecia opened. On each canker, three individual aecia were sampled separately by gently rupturing the aecial structure with a sterile scalpel tip and transferring the released aeciospores into sterile 1.5 mL microcentrifuge tubes [11]. All samples were placed in a desiccator containing a silica-based desiccant, lyophilized, and stored at −80 °C.

### 2.2. DNA Extraction and Genome Sequencing

Total genomic DNA was extracted using a CTAB method following reference [12]. DNA quality was examined by 1% agarose gel electrophoresis to evaluate integrity. Purity was measured using a NanoPhotometer^®^ spectrophotometer (Implen, Munich, Germany), while DNA concentration was determined with a Qubit fluorometer (Thermo Fisher Scientific, Suzhou, China). For Illumina sequencing, a paired-end library with an insert size of 400 bp was constructed using the TruSeqTM DNA Sample Prep Kit (Illumina, San Diego, CA, USA) following the supplier’s guidelines, and sequencing was carried out on the Illumina NovaSeq platform (2 × 150 bp). PacBio long-read sequencing libraries were also sequenced on the PacBio Revio platform, with raw long reads basecalled using Guppy under default settings to obtain high-quality data. Following the previously described method [13], Hi-C libraries were generated using the TruSeq DNA PCR-free kit (Illumina, San Diego, CA, USA) and sequenced on the Illumina NovaSeq platform (2 × 150 bp). Quality control of PacBio long reads was performed using Chopperv1.5.0 (http://gitcode.com/gh_mirrors/cho/chopper, accessed on 26 September 2025). Illumina short reads were processed with Fastp to remove low-quality sequences [14].

### 2.3. Genome Assembly and Assessment

The genome size of *C. ribicola* LQ was first inferred from a 19-mer distribution using jellyfish software (v2.0.0) based on Illumina paired-end reads [15]. For de novo genome assembly, PacBio long reads corrected with Falcon (v1.8.7) were assembled to generate an initial assembly by NextDenovo (v2.5.2) with the following parameters: read cutoff = 10 k, genome size = 300 m, pa correction = 10 [16]. To improve assembly accuracy and completeness, three successive rounds of error correction were conducted using Pilon (v1.23), which incorporated high-quality Illumina short-read data under default parameters [17]. HapHiC software v1.0.3 was employed to cluster, order, and orient the assembled contigs or scaffolds, thereby generating a chromosome-level genome assembly. Hi-C interaction maps were subsequently used to identify and correct potential assembly inconsistencies. Genome completeness was evaluated using the default parameters of Benchmarking Universal Single-Copy Orthologs (BUSCO) v5.4.5 [18] and the fungi_odb12 database.

### 2.4. Component Prediction

Gene prediction employed multiple tools, including Augustus v2.5.5 [19], GlimmerHMM v3.0.4 [20], and GeneMark-ES v4.17 [21]. Homology prediction was performed using exonerate software v2.2.0 (http://www.ebi.ac.uk/about/vertebrate-genomics/software/, accessed on 3 October 2025). The prediction results were integrated using EVidenceModeler software v2.0.0 [22]. Using RepeatModeler v1.0.8 (http://www.repeatmasker.org/RepeatModeler/, accessed on 11 October 2025) and RepeatScout v1.0.5 (http://repeatscout.bioprojects.org/, accessed on 14 October 2025), a de novo repeat library was generated and subsequently compared with the Swiss-Prot database. The combined repeat library was subsequently subjected to genome-wide repeat annotation using RepeatMasker v4.1.4. In parallel, a suite of complementary bioinformatics tools was employed to identify and characterize non-coding RNAs within the *C. ribicola* LQ genome, including Barrnap 0.9 (https://github.com/tseemann/barrnap, accessed on 23 October 2025) for rRNA prediction, tRNAscan-SE (http://lowelab.ucsc.edu/tRNAscan-SE/, accessed on 26 October 2025) for identifying tRNA regions and secondary structures, and cmscan (with default parameters) for detecting sRNAs and miRNAs by aligning sequences to the Rfam database (http://rfam.xfam.org/, accessed on 29 October 2025).

### 2.5. Genome Annotation

Functional annotation of the predicted gene set was performed using DIAMOND (v2.0.14) software by sequence similarity searches against several public repositories, including the National Center for Biotechnology Information (NCBI) Non-Redundant (NR), Kyoto Encyclopedia of Genes and Genomes (KEGG), EuKaryotic Orthologous Groups (KOG), Gene Ontology (GO), and Swiss-Prot databases, to infer potential gene functions. Protein structural domains were further identified using PfamScan (v1.6) based on the Pfam database. Putative carbohydrate-active enzymes (CAZymes) in the *C. ribicola* LQ genome were annotated using hmmscan (v3.2.1; http://hmmer.org/, accessed on 6 November 2025), with an e-value cutoff of <1 × 10^−5^ and a sequence coverage threshold of at least 70%. The resulting CAZyme candidates were further classified according to the CAZy database (http://www.cazy.org, accessed on 14 November 2025) based on their functional module annotations. BLAST software was used to predict genes associated with antibiotic resistance against an antibiotic resistance database. The database of fungal virulence factors was used to predict virulence factors within the genome. SignalP 5.0 software (http://www.cbs.dtu.dk/services/SignalP/, accessed on 23 November 2025) was employed to predict signal peptide sequences in protein-coding genes. TMHMM (Server v 2.0) was used to predict transmembrane helix structures in protein-coding genes [23]. TargetP 2.0 software was used to analyze protein subcellular localization. EffectorP 3.0 software was used to predict effector proteins of the pathogenic fungus. For cytochrome P450 family classification in *C. ribicola* LQ, all protein sequences were aligned against the fungal cytochrome P450 database using BLAST v2.10.1 [24]. The resulting hits were annotated following the nomenclature provided by the cytochrome P450 database (https://cyped.biocatnet.de/, accessed on 20 December 2025), with an e-value threshold of ≤1 × 10^−5^ applied throughout the analysis. Perform protein sequence alignment in the Transporter Classification Database (TCDB) using the BLAST method. This study did not involve the use of custom scripts or customized command lines. All comparative analyses were conducted using publicly available software. For any software tools where specific parameters are not specified, default settings were applied.

## 3. Results and Discussions

### 3.1. Genome Sequencing and Assembly

Illumina sequencing generated 19.14 Gb of high-quality clean data, while Hi-C sequencing produced 50.13 Gb of clean reads. In addition, PacBio sequencing yielded 22.4 Gb of HiFi reads (Table S1).

The 19.14 Gb of high-quality Illumina sequencing data were subjected to 19-mer analysis to construct a depth distribution profile of the sequencing reads (Figure S1). Based on the k-mer analysis, the genome size of *C. ribicola* LQ was estimated to be approximately 304.43 Mb, with a heterozygosity rate of about 0.57%. The k-mer distribution curve displayed two prominent peaks within the depth range of 20–60, with the second peak exhibiting a noticeably higher abundance than the first. The two major peaks observed in the k-mer distribution suggest a high level of heterozygosity, which is consistent with the known dikaryotic nature of rust fungi [25,26].

**Figure 2:**
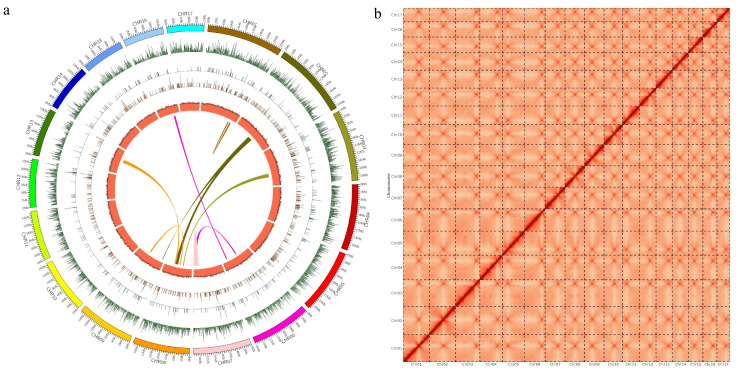
Genome assembly and features of *C. ribicola* LQ. (**a**): Circos plot illustrating genomic features of *C. ribicola* LQ. From the outermost circle to the innermost tracks: gene density, CAZy, fungal virulence factors, GC ratio, and multicollinearity; (**b**): Hi-C heatmap showing chromatin interaction intensity across assembled pseudo-chromosomes.

Based on the HiFi reads, we have completed the preliminary assembly of the *C. ribicola* LQ genome. The genome comprised 184 contigs, and its size was 262.5 Mb. The N50 was 4.22 Mb for the *C. ribicola* LQ genome, and the guanine-cytosine (GC) content percentage was 38.4% (Table 1). Finally, the genome with 17 chromosomes was generated, with size of 262.51 Mb, and scaffolds N50 lengths of 15.40 Mb (Figure 2a and Table 1). The high concordance observed between the chromosome-level genome assembly and the Hi-C interaction maps suggests that the assembled genome is of high accuracy and reliability (Figure 2b). A draft genome of *C. ribicola* strain 11-2 was released in 2014 using Illumina short-read sequencing [9]. In 2022, the Cypress_4 v1.0 genome was published following sequencing with PacBio technology and assembly using Flye; the genome size was 211.63 Mb, and the scaffold N50 was only 31 kb (comprising 214 scaffolds) [10]. The 11-2 and the Cypress_4 v1.0 assembly are considerably smaller than our assembly, likely reflecting the limitations of short-read-only approaches in resolving the highly repetitive nature of the *C. ribicola* genome.

Furthermore, the Benchmarking Universal Single-Copy Orthologs (BUSCOs) v5.4.5 were used to assess the assembly quality. The evaluation yielded the following BUSCO statistics: 94.83% complete, 94.03% complete single-copy, 0.8% duplicated, 2.5% fragmented, and 2.67% missing (Table S2). These results demonstrate that the *C. ribicola* LQ genome sequence had high completeness and contiguity.

It is worth noting that the k-mer-based genome size estimation was approximately 13.5% larger than the final assembled genome size. This discrepancy is commonly observed in fungal genome assemblies, particularly for species with high repeat content [27,28]. Several factors may account for this difference. First, highly repetitive regions, including transposable elements (TEs) which constitute 7–10% of typical fungal genomes and can be substantially higher in rust fungi, often resist assembly due to read length limitations and algorithmic challenges in distinguishing near-identical repeats [27]. Second, the dikaryotic nature of *C. ribicola* presents a unique assembly challenge. In dikaryotic fungi, each cell contains two distinct haploid nuclei, and the heterozygosity between haplotypes can lead to the retention of both haplotypes as separate contigs (haplotigs), which artificially inflates the estimated genome size while complicating the assembly process [29]. Hi-C-based scaffolding may inadvertently filter or collapse certain allelic regions during chromosome assignment [30]. Third, subtelomeric and centromeric regions, known to be repeat-rich and AT-rich, are frequently underrepresented in final assemblies [31]. Therefore, an unassembled sequence most likely corresponds to highly repetitive genomic compartments, including TEs, subtelomeric regions, and haplotype-specific sequences. Addressing these regions would require Oxford Nanopore ultra-long reads or telomere-to-telomere assembly strategies, which remain challenging for complex fungal genomes at present. Future work focusing on these unassembled regions may provide additional insights into the evolutionary dynamics and host adaptation mechanisms of *C. ribicola*.

### 3.2. Genome Component of C. ribicola LQ

#### 3.2.1. Gene Prediction

In the *C. ribicola* LQ genome, a total of 10,222 coding genes with an average length of 1708.5 bp were predicted. The cumulative length of these genes amounted to 17.46 Mb, accounting for 6.51% of the genome. The average number of exons per gene was 4.9. The total length and average length of the CDS were 12.97 Mb and 1268.7 bp, respectively (Table S3).

#### 3.2.2. Repeat Sequences Prediction

Repetitive sequences are ubiquitous features of eukaryotic genomes and represent evolutionarily ancient genomic components. They are generally classified into two main groups: interspersed repeats (IRs) and tandem repeats (TRs) [32]. TRs mainly consist of microsatellites, minisatellites, and satellite DNA sequences. In contrast, IRs are primarily composed of retrotransposons and DNA transposons [33]. Repetitive sequences in the *C. ribicola* LQ genome were characterized using a combination of homology-based and de novo prediction approaches. A total of 250.15 Mb of repeat sequences were detected, representing approximately 91.67% of the entire genome. Among these, unclassified repeats were the most abundant repetitive components (74.36%), while other repetitive sequences included retroelements (6.41%), long terminal repeats (LTRs; 6.2%) and Gypsy/DIRS1 (4.68%) also making a significant contribution (Table S4). Repetitive sequences are essential for chromosome architecture, regulation of gene expression, genome stability, and evolutionary processes. Therefore, comprehensive characterization of genomic repeat sequences is of great importance for advancing genome-wide research [34].

#### 3.2.3. Non-Coding RNA Prediction

The non-coding RNAs predicted in the *C. ribicola* LQ genome are summarized in Table S5. A total of 180 ncRNAs were identified in the *C. ribicola* LQ genome, including 127 tRNAs, 25 rRNAs (3 5S, 5 5.8S, 8 28S, and 18 18S), 3 miRNAs, 4 CD boxes, and 21 other ncRNAs.

### 3.3. Subsystem Analysis

#### 3.3.1. CAZymes

**Figure 3:**
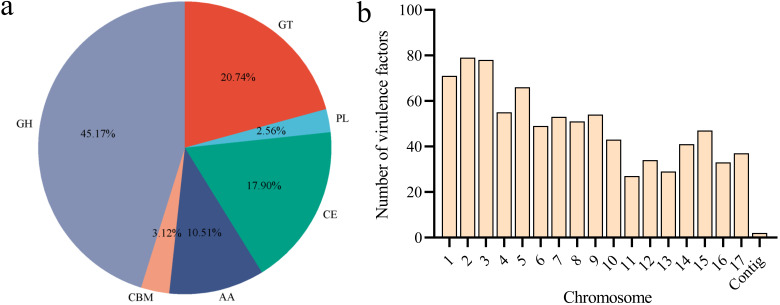
CAZymes and fungal virulence factors in *C. ribicola* LQ. (**a**): CAZymes in *C. ribicola* LQ; (**b**): Analysis of fungal virulence factors of *C. ribicola* LQ. Contig: Sequences not anchored to chromosome.

Complex carbohydrates are abundant in natural systems and perform a variety of essential biological functions, such as providing structural support, serving as energy reserves, and facilitating cellular recognition both within and among organisms. The synthesis, modification, and degradation of these carbohydrate are mediated by carbohydrate-active enzymes (CAZymes) [35]. A total of 352 CAZyme genes were identified from the genome of *C. ribicola* LQ, and these were distributed across 103 CAZyme families. Among these, glycoside hydrolases (GHs) represented the largest category, comprising 159 genes distributed across 48 families and accounting for 45.17% of the total CAZymes. Additional CAZyme classes included 73 glycosyl transferases (GTs, 31 families, 20.74%), 9 polysaccharide lyases (PLs, 6 families, 2.56%), 63 carbohydrate esterases (CEs, 8 families, 17.90%), 37 auxiliary activity enzymes (AAs, 12 families, 10.51%), and 11 carbohydrate-binding modules (CBMs, 7 families, 3.12%) (Figure 3a and Table S6).

#### 3.3.2. Antibiotic Resistance Genes

The development of antibiotic resistance is often driven by the horizontal transfer of antibiotic resistance genes (ARGs) among microorganisms [36]. Previous studies have extensively examined the mobilization and spread of particular ARGs, including the *cmx* genes [37], *ctx-m* genes [38], and the vancomycin-resistance operon [39]. The results indicated that 3 antibiotic resistance genes were predicted in the genome of *C. ribicola* LQ, accounting for 0.029% of the total protein-coding genes (Table S7). These genes may confer resistance to specific antibiotics.

#### 3.3.3. Fungal Virulence Factors

Virulence factors, including effectors, mycotoxins, cell wall–degrading enzymes, and organic acids, contribute in diverse ways depending on the infection mode, enabling pathogens to manipulate and colonize living plant tissues [40]. A total of 849 fungal virulence factors were predicted in the genome of *C. ribicola* LQ. Among these, the highest number of fungal virulence factors was located on chromosome 2 (79 genes, 9.31%), followed by chromosome 3 (78 genes, 9.19%), while the lowest number was located on chromosome 11 (27 genes, 3.18%) (Figure 3b and Table S8).

### 3.4. Subcellular Localization Analysis of Protein-Coding Genes

#### 3.4.1. Secretory Proteins

**Figure 4:**
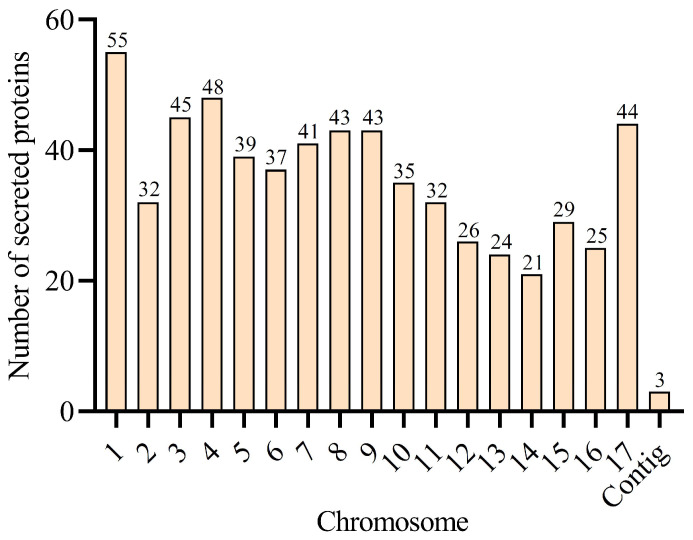
Secreted proteins in the genome of *C. ribicola* LQ. Contig: Sequences not anchored to chromosome.

In recent years, a growing number of small secreted proteins (SSPs) have been characterized in biotrophic filamentous plant pathogens, where they are recognized as key effectors involved in host–pathogen interactions. For instance, genome analysis of *Melampsora larici-populina* has identified 1184 genes encoding SSPs in this rust fungus [41]. A total of 622 secreted proteins were predicted in the genome of *C. ribicola* LQ (Table S9). The number of secreted proteins distributed across each chromosome ranges from 21 to 55 (Figure 4). The quantity of secreted proteins in this organism was significantly lower than that of *M. larici-populina*.

#### 3.4.2. Secondary Metabolites

**Figure 5:**
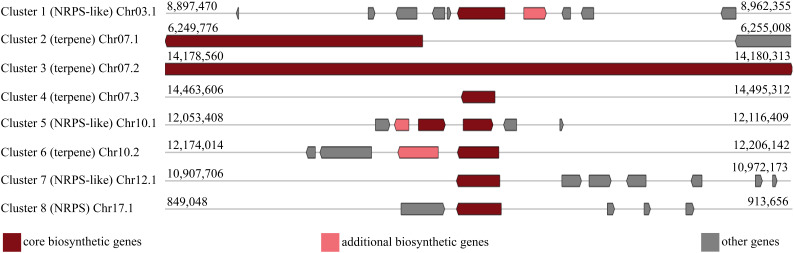
Secondary metabolite gene clusters of *C. ribicola* LQ.

Fungi produce a broad spectrum of secondary metabolites during their growth, which constitute an important sources of bioactive substances. These metabolites encompass terpenes, nonribosomal peptides, vitamins, and other biologically active molecules. Many of these secondary metabolites display a wide range of physiological effects in biomedical research, such as lipid-lowering activity, antitumor properties, immune modulation, and metabolic regulation [42,43]. Terpenes are polymers derived from isoprene units and typically form complex polycyclic structures; they may undergo further chemical modifications to generate terpenoids. Both terpenes and terpenoids are often associated with characteristic sensory properties, including aroma, flavor, or pigmentation. Functionally, terpenoid metabolites can act either as phytotoxins, such as fusicoccin A produced by *Diaporthe amygdali*, or as growth-promoting regulators, such as gibberellin GA14 from *Fusarium fujikuroi* [44]. In addition, nonribosomal peptides are synthesized independently of mRNA templates by large multi-enzyme complexes known as nonribosomal peptide synthetases (NRPSs). These peptides frequently contain noncanonical amino acids and often undergo further enzymatic modifications. Representative NRPS-derived compounds include β-lactam antibiotics, cyclosporine A and echinocandins [45]. A total of 8 clusters associated with secondary metabolites were predicted in the genome of *C. ribicola* LQ, including four terpene clusters, three NRPS-like clusters, and one NRPS cluster (Figure 5).

#### 3.4.3. Effector Proteins

Fungal effector proteins are typically characterized as small secreted proteins containing an N-terminal signal peptide and lacking transmembrane domains or other known targeting sequences [46,47,48]. To facilitate successful infection, plant pathogens deliver effector proteins into host tissues, where they interfere with and reprogram host cellular processes [49]. To date, various effector proteins have been identified in rust fungi—such as AvrM, AvrL567, AvrP123, and AvrP4 in *Melampsora lini*; the rust-transferred protein RTP1 in *Uromyces fabae*; and PGTAUSPE-10-1 in *Puccinia graminis* f. sp. *Tritici* [50,51,52]. A total of 4276 effector proteins were predicted. Among them, 3734 were identified as cytoplasmic effectors (87.32%), 333 as apoplastic effectors (7.79%), and 213 as cytoplasmic/apoplastic effectors (4.89%) (Figure S2 and Table S10). The functions of these effector proteins require further experimental validation.

### 3.5. Annotation of Gene Function

The analysis results indicate that 9028, 6001, 5200, and 3231 annotated genes were obtained from the Nr, KOG, Swiss-Prot, and KEGG databases, respectively (Table S11). On the basis of the resulting annotation information, additional analyses were carried out to further investigate the genome of *C. ribicola* LQ.

#### 3.5.1. GO Annotation

**Figure 6:**
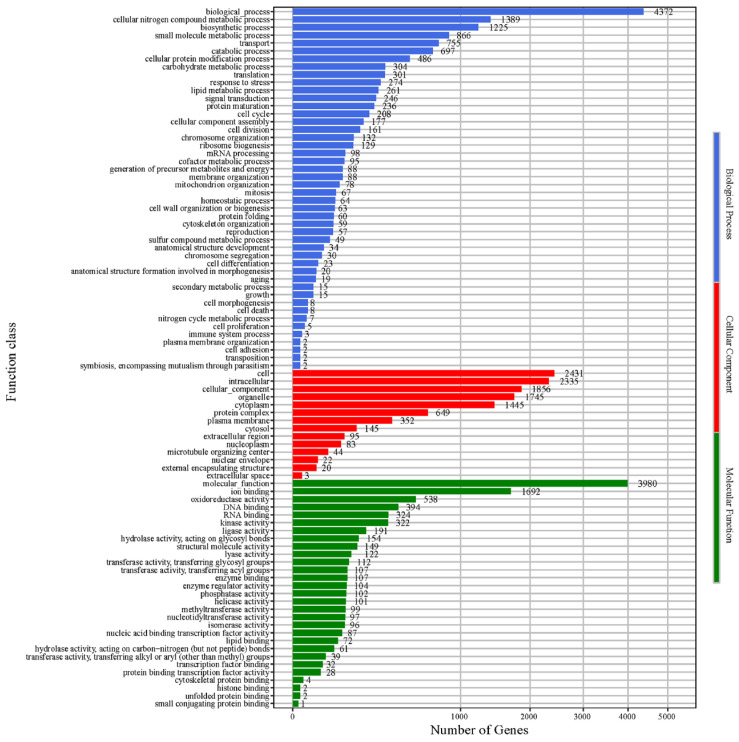
The GO function annotation of *C. ribicola* LQ.

A total of 4830 genes were annotated in the GO database. The GO terms in the Biological Process category were the most common and included “biological process” (4372 genes), “cellular nitrogen compound metabolic process” (1389 genes), and “biosynthetic process” (1225 genes); within the Cellular Component category, the most frequently detected terms were “cell” (2431 genes), “intracellular” (2335 genes), and “cellular component” (1856 genes); and within the Molecular Function category, the most common GO terms were “molecular function” (3980 genes), “ion binding” (1692 genes), and “oxidoreductase activity” (538 genes) (Figure 6). GO annotation of the *C. ribicola* Cypress_4 v1.0 genome identified 4684 genes [10], slightly fewer than the 4830 genes annotated in the genome of *C. ribicola* LQ, this difference may be attributable to variations in the annotation software and pipelines.

#### 3.5.2. KEGG Annotation

**Figure 7:**
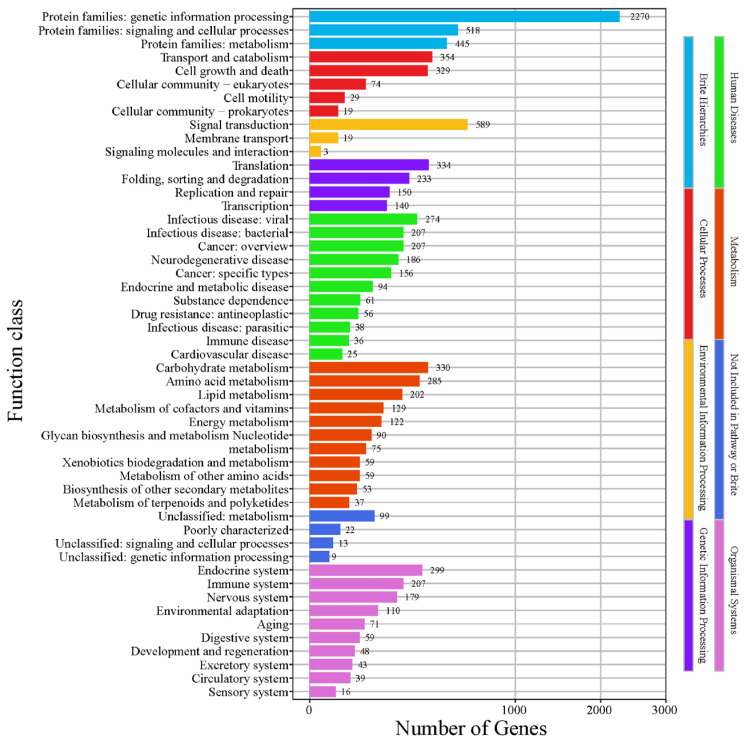
The KEGG function annotation of *C. ribicola* LQ.

A total of 3231 genes were successfully mapped to the KEGG database, representing 31.61% of the predicted gene. These genes were classified into eight major categories: Metabolism (11 branches, 1441 genes), Genetic Information Processing (4 branches, 857 genes), Environmental Information Processing (3 branches, 611 genes), Cellular Processes (5 branches, 805 genes), Organismal Systems (10 branches, 1071 genes), Brite Hierarchies (3 branches, 3233 genes), Not Included in Pathway or Brite (4 branches, 143 genes), and Human Diseases (11 branches, 1340 genes). Within the Metabolism category, the 1441 genes were further classified into 11 subcategories, primarily “Carbohydrate metabolism” (330 genes), “Amino acid metabolism” (285 genes), “Lipid metabolism” (202 genes), “Energy metabolism” (122 genes), “Xenobiotics biodegradation and metabolism” (59 genes), “Metabolism of cofactors and vitamins” (129 genes), “Biosynthesis of other secondary metabolites” (53 genes), “Metabolism of other amino acids” (59 genes), “Metabolism of terpenoids and polyketides” (37 genes), “Nucleotide metabolism” (75 genes), and “Glycan biosynthesis and metabolism” (90 genes) (Figure 7). This is largely consistent with the KEGG annotation results for the *C. ribicola* Cypress_4 v1.0 genome [10]; “carbohydrate metabolism” and “amino acid metabolism” are the most abundant categories, reflecting the pathogen’s robust capabilities for carbohydrate degradation and amino acid utilization during host infection.

#### 3.5.3. KOG Annotation

KOG functional annotation results are presented in Figure S3. In total, 6001 genes were assigned to the KOG database, accounting for 58.71% of the predicted gene. Functional classification indicated that these genes were distributed across multiple categories, including “Function unknown” (1581 genes), “Posttranslational modification, protein turnover, chaperones” (527 genes), “Carbohydrate transport and metabolism” (436 genes), “Signal transduction mechanisms” (359 genes), “Translation, ribosomal structure and biogenesis” (355 genes), “Intracellular trafficking, secretion, and vesicular transport” (346 genes), “RNA processing and modification” (284 genes), and “Lipid transport and metabolism” (274 genes) (Figure S3). This is similar to the total number of genes with KOG annotations in the *C. ribicola* Cypress_4 v1.0 genome (5929 genes) [10], indicating comparable annotation coverage.

#### 3.5.4. TCDB Annotation

The Transporter Classification Database (TCDB)-based annotation results are presented in Figure S4. In total, 1204 genes were successfully assigned to the TCDB database, representing 11.78% of the predicted gene. The results indicated that these genes were classified into seven class, including “Primary Active Transporters” (287 genes), “Electrochemical Potential-driven Transporters” (271 genes), “Channels; Pores” (223 genes), “Accessory Factors Involved in Transport” (201 genes), “Incompletely Characterized Transport Systems” (187 genes), “Group Translocators” (26 genes), and “Transmembrane Electron Carriers” (9 genes) (Figure S4a, Table S12). These genes were further subdivided into 24 subclass, primarily including “Porters” (267 genes), “P-P-bond-hydrolysis-driven transporters” (246 genes), and “Auxiliary transport proteins” (199 genes) (Figure S4b).

## 4. Conclusions

In this study, we report the genomic data of *C. ribicola* LQ generated through the integration of multiple sequencing platforms. Gene function annotation was performed using a range of public databases to comprehensively characterize the genome. The resulting assembly, together with its annotation resources, constitutes the first chromosome-level genome of *C. ribicola*. These genomic data provide a valuable foundation for future investigations into species evolution and phylogenetic relationships at the genome level. Moreover, this study provides key genomic resources for understanding the pathogenic mechanisms, host interactions, and resistance gene discovery of *C. ribicola*, laying the foundation for the development of virulence-targeted control strategies.

## Figures and Tables

**Table 1:** Genome assembly features of *C. ribicola* LQ.

Contents	Contigs	Scaffolds
Total sequence number	184	95
Total sequence length	262.50 Mb	262.51 Mb
Min sequence length	5 kb	15.77 kb
Max sequence length	13.77 Mb	20.59 Mb
N20	7.33 Mb	19.22 Mb
N20 Number	6	3
N50	4.22 Mb	15.40 Mb
N50 Number	20	8
N90	1.32 Mb	11 Mb
N90 Number	64	16
*N* rate	0	3.85 × 10^−5^
GC content %	38.4	38.4
Sequences greater than 1 kb	184	95

## Data Availability

All sequencing data, including the raw Illumina short reads, PacBio long reads, and Hi-C sequencing, have been deposited in the Genome Sequence Archive (GSA [53]) in the National Genomics Data Center [54] under BioProject PRJNA1405245. The Illumina raw data are available under accession SRR36889336 [55], the PacBio raw data under SRR36889335 [56], and the Hi-C raw data under SRR36889334 [57]. The assembled genome and the annotation data have been deposited into the Figshare repository [58,59].

## Supplementary Materials

The following supporting information can be downloaded at: https://www.mdpi.com/article/10.3390/jof12070471/s1, Figure S1: Genome survey based on 19-mer frequency distribution of base error-corrected reads; Figure S2: Identification of effector proteins of *C. ribicola* LQ; Figure S3: The KOG function annotation of *C. ribicola* LQ; Figure S4: The TCDB function annotation of *C. ribicola* LQ; Table S1: Statistics for the sequencing data of the *C. ribicola* LQ genome; Table S2: Statistics from the BUSCO analysis of the *C. ribicola* LQ genome; Table S3: Characteristics of the gene prediction of *C. ribicola* LQ; Table S4: Statistics of the *C. ribicola* LQ repetitive sequence prediction results; Table S5: Statistical results of the non-coding RNAs in *C. ribicola* LQ; Table S6: Carbohydrate-active enzyme annotation results; Table S7: Antibiotic resistance gene in *C. ribicola* LQ; Table S8: Virulence factors gene in *C. ribicola* LQ; Table S9: Secretory proteins in *C. ribicola* LQ; Table S10: Effector proteins in *C. ribicola* LQ; Table S11: Statistics of *C. ribicola* LQ protein-coding gene annotation; Table S12: TCDB annotation statistics for *C. ribicola* LQ.
