# Supplementary material for: Chromosome-Level Genome Assembly and Annotation of the Cronartium ribicola Strain LQ, an Important Fungal Forest Pathogen from China
Source: J Fungi (Basel). 2026 Jun 26;12(7):471. doi: 10.3390/jof12070471 (PMC13412462; doi:10.3390/jof12070471)
Supplement: Supplementary file 1 [file jof-12-00471-s001.zip › Chart Description.pdf]

Figure 1: Disease symptoms caused by *Cronartium ribicola* strain LQ in the field

a: *Pinus armandii* infected with *C. ribicola* LQ; b: Morphology of *C. ribicola* LQ under scanning electron microscopy. The aeciospores are ovoid, with a verrucose surface ornamentation.

Figure 2: Genome assembly and features of *C. ribicola* LQ

a: Circos plot illustrating genomic features of *C. ribicola* LQ. From the outermost circle to the innermost tracks: gene density, CAZy, fungal virulence factors, GC ratio, and multicollinearity; b: Hi-C heatmap showing chromatin interaction intensity across assembled pseudo-chromosomes.

Figure 3: CAZymes and fungal virulence factors in *C. ribicola* LQ

a: CAZymes in *C. ribicola* LQ; b: Analysis of fungal virulence factors of *C. ribicola* LQ. Contig: Sequences not anchored to chromosome.

Figure 4: Secreted proteins in the genome of *C. ribicola* LQ

Contig: Sequences not anchored to chromosome.

Figure 5: Identification of secondary metabolite gene clusters of *C. ribicola* LQ

Figure 6: The GO function annotation of *C. ribicola* LQ

Figure 7: The KEGG function annotation of *C. ribicola* LQ

Figure S1: Genome survey based on 19-mer frequency distribution of base error-corrected reads

Figure S2: Identification of effector proteins of *C. ribicola* LQ

Figure S3: The KOG function annotation of *C. ribicola* LQ

Figure S4: The TCDB function annotation of *C. ribicola* LQ

Table 1: Genome assembly features of *C. ribicola* LQ

Table S1: Statistics for the sequencing data of the *C. ribicola* LQ genome

Table S2: Statistics from the BUSCO analysis of the *C. ribicola* LQ genome

Table S3: Characteristics of the gene prediction of *C. ribicola* LQ

Table S4: Statistics of the *C. ribicola* LQ repetitive sequence prediction results

Table S5: Statistical results of the non-coding RNAs in *C. ribicola* LQ

Table S6: Carbohydrate-active enzyme annotation results

Table S7: Antibiotic resistance gene in *C. ribicola* LQ

Table S8: Virulence factors gene in *C. ribicola* LQ

Table S9: Secretory proteins in *C. ribicola* LQ

Table S10: Effector proteins in *C. ribicola* LQ

Table S11: Statistics of *C. ribicola* LQ protein-coding gene annotation

Table S12: TCDB annotation statistics for *C. ribicola* LQ
