# Supplementary figures and images for: Chromosome-Level Genome Assembly and Annotation of the Cronartium ribicola Strain LQ, an Important Fungal Forest Pathogen from China
Source: J Fungi (Basel). 2026 Jun 26;12(7):471. doi: 10.3390/jof12070471 (PMC13412462; doi:10.3390/jof12070471)

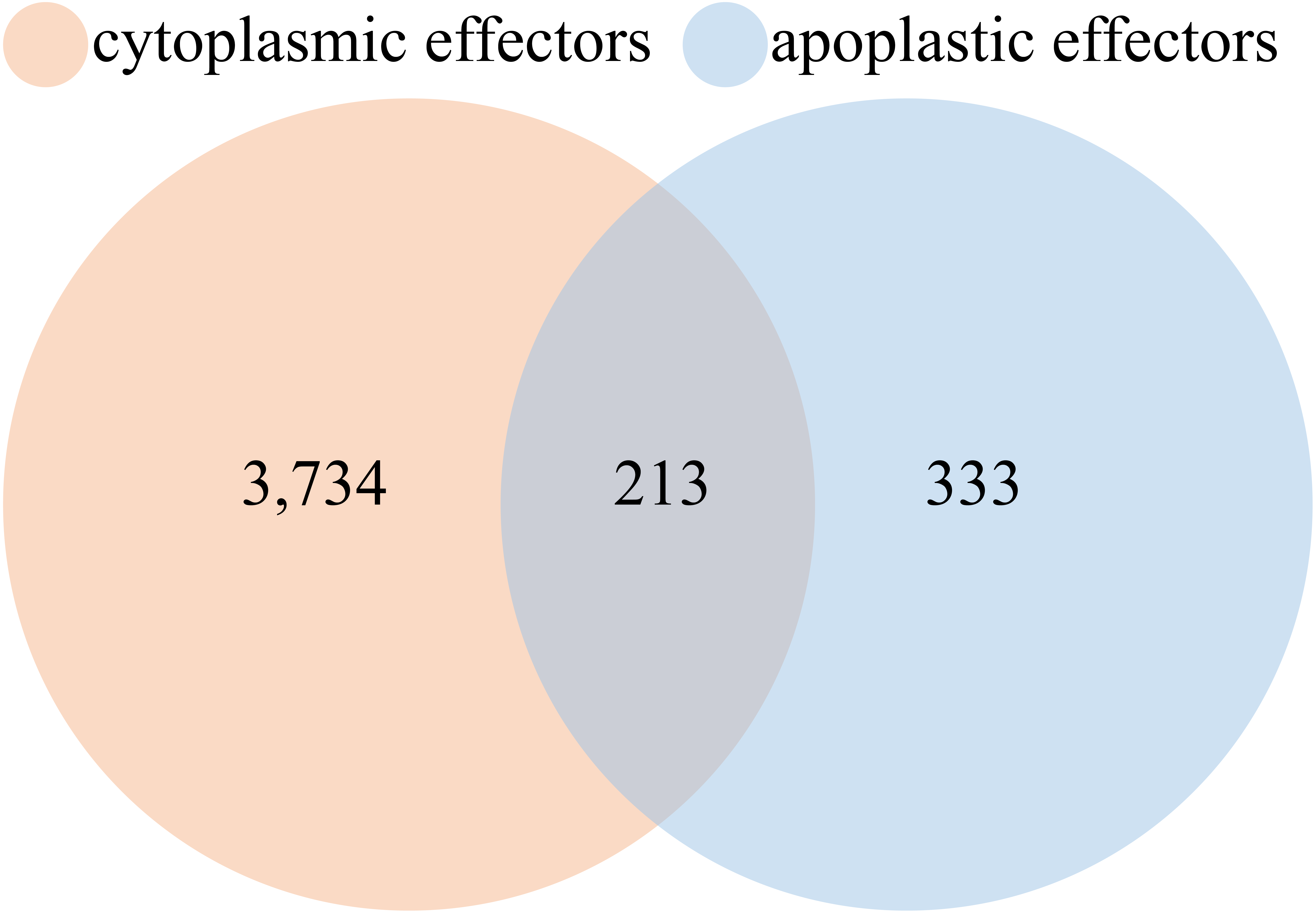

Supplement: Supplementary file 1 [file jof-12-00471-s001.zip › Figure S2.png]

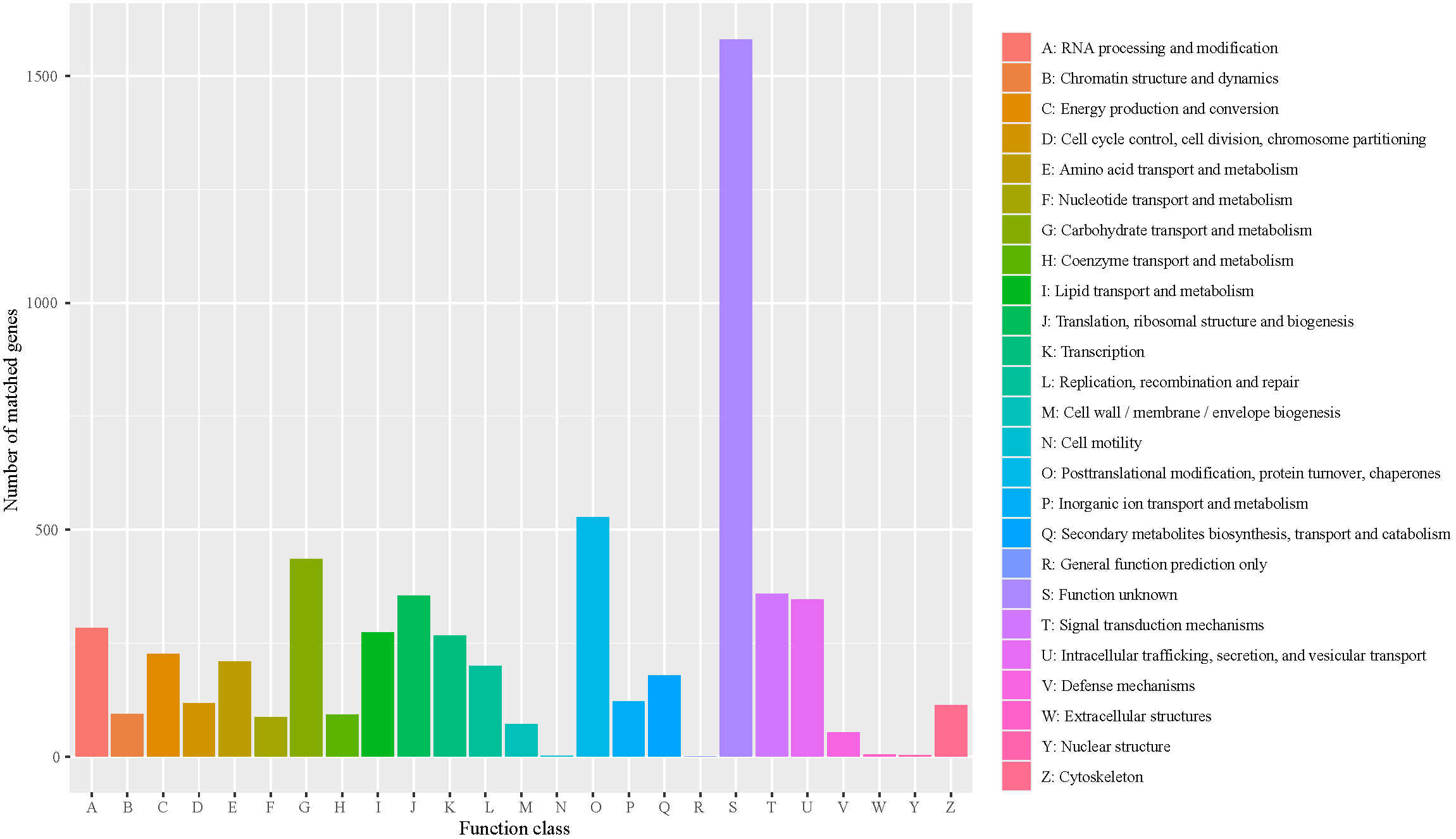

Supplement: Supplementary file 1 [file jof-12-00471-s001.zip › Figure S3.png]
